# Chemokine receptors in the rheumatoid synovium: upregulation of CXCR5

**DOI:** 10.1186/ar1475

**Published:** 2004-12-16

**Authors:** Caroline Schmutz, Alison Hulme, Angela Burman, Mike Salmon, Brian Ashton, Christopher Buckley, Jim Middleton

**Affiliations:** 1 Leopold Muller Arthritis Research Centre, Robert Jones and Agnes Hunt Orthopaedic Hospital, Oswestry, UK; 2 Division of Immunity and Infection, Medical Research Council Centre for Immune Regulation, University of Birmingham, Edgbaston, UK; 3 Institute for Science and Technology in Medicine, Medical School, Keele University, Stoke-on-Trent, UK

**Keywords:** chemokine receptors, CXCR5, microarrays, rheumatoid synovium

## Abstract

In patients with rheumatoid arthritis (RA), chemokine and chemokine receptor interactions play a central role in the recruitment of leukocytes into inflamed joints. This study was undertaken to characterize the expression of chemokine receptors in the synovial tissue of RA and non-RA patients. RA synovia (*n *= 8) were obtained from knee joint replacement operations and control non-RA synovia (*n *= 9) were obtained from arthroscopic knee biopsies sampled from patients with recent meniscal or articular cartilage damage or degeneration. The mRNA expression of chemokine receptors and their ligands was determined using gene microarrays and PCR. The protein expression of these genes was demonstrated by single-label and double-label immunohistochemistry. Microarray analysis showed the mRNA for *CXCR5 *to be more abundant in RA than non-RA synovial tissue, and of the chemokine receptors studied CXCR5 showed the greatest upregulation. PCR experiments confirmed the differential expression of *CXCR5*. By immunohistochemistry we were able to detect CXCR5 in all RA and non-RA samples. In the RA samples the presence of CXCR5 was observed on B cells and T cells in the infiltrates but also on macrophages and endothelial cells. In the non-RA samples the presence of CXCR5 was limited to macrophages and endothelial cells. *CXCR5 *expression in synovial fluid macrophages and peripheral blood monocytes from RA patients was confirmed by PCR. The present study shows that CXCR5 is upregulated in RA synovial tissue and is expressed in a variety of cell types. This receptor may be involved in the recruitment and positioning of B cells, T cells and monocytes/macrophages in the RA synovium. More importantly, the increased level of CXCR5, a homeostatic chemokine receptor, in the RA synovium suggests that non-inflammatory receptor–ligand pairs might play an important role in the pathogenesis of RA.

## Introduction

Rheumatoid arthritis (RA) is a chronic inflammatory condition that affects multiple joints, and it results in the accumulation of leukocytes within the synovial tissue (ST) and synovial fluid (SF). The inflammatory infiltrate consists predominantly of B lymphocytes, T lymphocytes and macrophages in the ST, whereas neutrophils are mainly found in the SF. The lymphocyte infiltration is organized in lymphoid-like microstructures in just under 50% of the RA patients; however, the patients present germinal centre reactions in only 20% of cases [1]. The pathogenesis of the RA is still largely unknown but leukocytes and their products play an important role in the development of inflammation, joint destruction and pain [2,3]. The attraction of leukocytes into the joints is controlled by chemokines, a family of small chemotactic cytokine-like molecules that act as potent mediators of inflammation [4].

Chemokine activity is dependent on the presence of and interaction with chemokine receptors on the leukocyte surface. Indeed, chemokines and their receptors are involved together in the development and perpetuation of inflammation [5]. *In vitro *and *in vivo *experiments have indicated that blocking chemokines or their receptors could potentially provide an effective treatment of inflammatory diseases [5,6]. The 19 receptors so far identified belong to a super-family of G-protein-coupled receptors with seven transmembrane domains [7]. Chemokine receptors have a regulatory effect on the maturation and traffic of leukocytes, and they are implicated in several disease states [8]. There have been several reports on chemokine receptor expression on T cells from RA ST, RA SF and RA peripheral blood (PB) [9-13]. The expression of some chemokine receptors on monocytes/macrophages, dendritic cells and neutrophils has also been reported [14-17], and the importance of the role of chemokine receptors in RA is emerging [18,19].

CXCR5 is a chemokine receptor highly expressed in recirculating B cells, in subsets of CD4^+ ^and CD8^+ ^T cells and monocytes [20,21]. It also has been identified on B-cell infiltrates in Sjogren's syndrome [22,23]. CXCR5 is involved in the immune-system homeostasis and in lymphoid organogenesis [24]. Several morphological and functional studies suggest that lymphoid neogenesis takes place in RA [1,25,26]. Furthermore, an important disturbance of follicle and germinal centre formation in the spleen and Peyer's patches is observed in CXCR5-deficient mice [27]. CXCL13, the unique ligand of CXCR5, is also involved in follicular homing, as observed in CXCL13-deficient mice [28].

In view of the role of chemokine receptors in leukocyte traffic, the aim of the present study was to compare their expression in inflamed and non-inflamed tissue to shed light on which chemokine receptors may be involved in the recruitment and retention of leukocytes in ST. We examined chemokine receptor expression in ST taken from RA and non-RA patients using microarray technology, RT-PCR and immunohistochemistry. The microarray and RT-PCR experiments demonstrated the differential expression of *CXCR5*, and immunohistochemistry showed that this receptor is expressed in B-cell and T-cell infiltrates, on macrophages and blood vessels. Our study identifies CXCR5 as a potentially interesting therapeutic target in RA and points to the use of antagonists to this receptor as a treatment strategy in the disease.

## Materials and methods

### Tissue and cell source

Tissue samples were obtained from patients with RA (*n *= 8) who fulfilled the American Rheumatism Association criteria for RA (Table 1). The patients' mean age was 59 ± 14.8 years with a male to female ratio of 1:8. The disease duration of six out of eight RA patients was over 10 years. ST was taken from these subjects at the time of total knee replacement. Non-RA patients (*n *= 9) had knee joint symptoms for suspected articular cartilage or meniscal damage (Table 1). Their mean age was 47.6 ± 6.8 years with a male to female ratio of 8:1. Except for one patient, the non-RA patients had knee complaints for 1 year or less. ST biopsies were obtained from these patients at the time of arthroscopy. All samples were taken with informed consent and ethical approval. The ST samples were taken from the suprapatellar pouch and the medial gutter, which is reported to provide representative sampling of synovial membrane pathology [29]. Synovia were cut into approximately 4 mm^3 ^pieces and were either snap frozen in isopentane and stored in liquid nitrogen or formalin fixed and paraffin embedded.

Monocytes/macrophages were isolated from the PB and SF of another four RA patients (Table 1). In brief, the blood and hyaluronidase-treated SF were centrifuged over a ficoll cushion (Amersham Biosciences, Chalfont St Giles, UK). The macrophages were isolated from the buffy coat layer (lymphocytes, macrophages) by adherence onto a glass dish.

### RNA extraction

Total RNA was extracted from frozen blocks of synovia or from isolated monocytes/macrophages using TRIreagent solution (Sigma, Poole, UK) according to the manufacturer's recommendation. The quantity recovered was determined by spectrophotometry and the integrity was assessed by gel electrophoresis. For microarray experiments, equal quantities (7 μg) of RNA from each RA or non-RA patient were pooled and the messenger RNA was extracted using the mRNA GeneElute Kit (Sigma). The quantity recovered was determined by fluorometry using SYBR Green II (Molecular Probes, Leiden, The Netherlands). RNA had to be pooled since only small biopsies could be obtained from non-RA patients.

### Microarray technology

The panorama human cytokine gene array (Sigma-Genosys, Pampisford, UK) was used. This array contains 375 different cDNAs including 16 chemokine receptors and 33 chemokines, each printed in duplicate on nylon membranes.

The probe labelling and hybridization were carried out according to the manufacturer's instructions. Briefly, ^33^P-radiolabelled cDNA probes were prepared from 0.5 μg mRNA (see earlier) using human cytokine cDNA labelling primers (Sigma-Genosys) and AMV reverse transcriptase at 42°C, and were purified on a Sephadex^® ^G-25 spin column (Sigma-Genosys). The arrays were hybridized for 17–18 hours at 65°C, washed and subjected to autoradiography for various lengths of time using Kodak BioMax MR X-ray film.

The intensity of hybridization signals was quantified using the ArrayVision, version 6.0, software (Imaging Research Inc., Haverhill, UK). The intensity of each spot was corrected for background levels using the 'corners between spots' (set to 3 pixels) with or without 'segmentation' protocols, and were normalized for differences in labelling using the average values of seven housekeeping genes: *β_2_-microglobulin*, *β-actin*, *cyclophilin A*, *glyceraldehyde-3-phosphate dehydrogenase*, *HLA-A 0201 heavy chain*, *human hypoxanthine phosphoribosyl transferase*, and *α-tubulin*. The remaining two housekeeping genes, *L19 *and *transferrin R*, were excluded because of signal saturation and differential expression, respectively. The software performs the normalization automatically.

### Reverse transcription-polymerase chain reaction

Total RNA aliquots from individual patients were reverse transcribed using oligo(dT_18_) (MWG Biotech, Ebersberg, Germany) and MMLV reverse transcriptase (Promega, Southampton, UK) at 37°C for 1 hour. The reactions were terminated at 70°C for 10 min and were diluted to 80 μl with H_2_O. For two of the non-RA patients no more RNA was available for RT-PCR following microarray analysis.

The PCR reactions were normalized against the ribosomal RNA *L27 *using specific primers (MWG Biotech) (Table 2). Appropriate cDNA dilutions were used subsequently for the RT-PCR reactions using specific primers for *CXCR5 *(MWG Biotech) (Table 2). Specific primers were designed from the published sequences. The number of cycles and the annealing temperature were optimized for each primer pair. The RT-PCR conditions were one cycle at 94°C for 3 min, 57°C for 1 min and 72°C for 1 min, *X *cycles at 94°C for 1 min, 57°C for 1 min and 72°C for 1 min, and one cycle at 94°C for 1 min, 57°C for 1 min and 72°C for 10 min. *X *equals 34 cycles for *CXCR5 *and 24 cycles for *L27*.

### Immunohistochemistry for CXCR5

The ST from the patients that had been examined at the transcription level was also available for protein expression analysis. Paraffin embedded sections were cut 4 μm thick on 3-aminopropyltriethoxysilane-coated slides. Sections were deparaffinized and rehydrated before blocking endogenous peroxidase activity with H_2_O_2 _(0.3%) in methanol. The slides were rinsed with PBS and incubated with normal serum (1:67 in PBS) for 10 min before applying anti-human CXCR5 monoclonal antibody (1:666; R&D, Abingdon, UK) and the respective IgG control (Dako, Ely, UK). The sections were rinsed with PBS and incubated with biotinylated secondary antibody. The antibody binding was detected using reagents in the Vectastain ABC Elite kit (Vector, Peterborough, UK) and the chromogen 3,3'-diaminobenzidine (DAB) (Vector). Sections were rinsed and counter stained in Mayer's haematoxylin.

B cells and macrophages were localized using anti-human CD20 antibodies (1:100; Dako) and CD68 antibodies (clone PG-M1, 1:75; Dako), respectively. CD20 required antigen demasking by 15 min microwaving in citrate buffer (pH 6.0), but H_2_O_2 _treatment was not necessary. CD68 antigen was demasked using 0.05% pronase in Tris-buffered saline (pH 7.2) for 10 min.

Double immunohistochemistry was performed with anti-human CD3 rabbit monoclonal antibodies (Labvision) and CXCR5 antibodies. The slides were deparaffinized, rehydrated and microwaved for 15 min in citrate buffer pH 6.0 before being treated with H_2_O_2 _in methanol. The slides were incubated with 2.5% normal swine serum for 20 min before applying CD3 diluted 1:60 in 2.5% serum for 30 min. The sections were rinsed with PBS and were incubated with swine anti-rabbit antibody linked to alkaline phosphatase (1:40; Dako). CD3 binding was detected using Vector Red substrate (Vector). Sections were rinsed and were either counter stained in Methyl Green (Vector) or subjected to a second round of immunohistochemistry. CXCR5 was used as for single immunohistochemistry except that blocking and antibody dilutions were made in 2.5% normal horse serum and CXCR5 was revealed with DAB-Nickel (Vector). No counter stain was performed for double immunohistochemistry sections.

## Results

### Patients and tissue selection

Synovia were obtained from knee joints as this allowed the use of arthroscopic samples of non-RA (normal) as controls instead of osteoarthritic tissue, which can show more enhanced inflammatory changes. The histology of H&E-stained RA synovial sections demonstrated classic signs of inflammation. Mononuclear cell infiltrates were visible in seven out of eight patients and consisted of aggregate structures; one of these seven patients also contained more germinal-like centre structures. In addition one patient revealed a diffuse infiltration. The synovium of the non-RA patients showed minimal signs of inflammation. In eight out of nine patients no mononuclear infiltrates were observed, and in one case only a small infiltrate was seen. No thickening of the intima was observed in the non-RA compared with the RA samples.

### Microarray analysis of chemokine receptor expression

To allow rapid preliminary screening of a large number of chemokines and their receptors in RA ST and non-RA ST, chemokine expression was investigated using microarrays.

**Figure 1:**
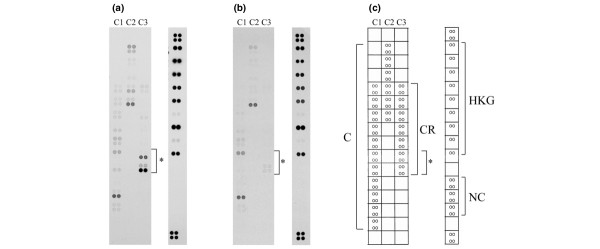
Microarray analysis of chemokine and chemokine receptor expression in the rheumatoid arthritis (RA) and non-RA synovia. A pair of human cytokine array membranes were hybridized to ^33^P-labelled cDNA probes prepared from pools of **(a) **RA mRNA (*n *= 8) and **(b) **non-RA mRNA (*n *= 9). The membranes were washed and autoradiographed. **(c) **The position of the 33 chemokines (C), the 16 chemokine receptors (CR), the nine positive control 'housekeeping genes' (HKG) and the six negative controls (NC). Each gene was printed in duplicate. The star indicates the position of the genes *CXCR1*, *CXCR2*, *CXCR4 *and *CXCR5 *(reading top to bottom) and shows their differential expression in RA and non-RA synovia. Exposure time was 7 days and 14 days for (a) and (b), respectively.

A pair of human cytokine microarrays including 16 chemokine receptors and 33 chemokines was hybridized with labelled cDNA probes prepared from mRNAs obtained from RA and non-RA pools of synovial RNA. Figure 1 shows the results of hybridization of the RA and non-RA probes to the array membranes. To reduce the bias that could be introduced during the quantification, arrays showing very similar signals for the housekeeping genes were chosen and only non-saturated and non-regulated signals/genes were used for normalization. The intensity of each spot was corrected for background levels. The analysis step was repeated eight times for each pair of autoradiogram.

Of the 16 chemokine receptors present, the expression of 12 chemokine receptors was visible on the RA microarrays. These were *CCR1*, *CCR2a*, *CCR5*, *CCR7*, *CCR9*, *CX3CR1*, *CXCR1*, *CXCR2*, *CXCR4*, *CXCR5*, *CXCR6 *(*STRL33*) and *Bob *(Table 3). Expression of the same receptors could be observed on the non-RA membranes with the exception of *Bob*, *CCR7 *and *CCR9*. *Bob*/*GPR15 *is an orphan receptor that is a coreceptor for human and simian immunodeficiency viruses, and its expression in the RA synovium is a novel observation that might be worthy of further investigation. The detection of *CCR7 *and *CCR9 *in RA was only possible after extended exposure times, but at the time points used for quantification no regulation was demonstrated. Four chemokine receptors (*CCR2b*, *CCR3*, *CCR4 *and *CCR6*) could not be detected in RA samples or non-RA samples under our conditions.

The most obvious differences between RA samples and non-RA samples were for the chemokine receptors *CXCR5 *and *CXCR2*, and to a lesser extent *CXCR4*, which gave stronger signals in RA samples (Fig. 1). In order to quantify the differential expression of these receptors the densities of autoradiographic spots were measured using ArrayVision software (Table 3). The criteria we set for a gene to be considered as upregulated or downregulated were a RA/non-RA ratio higher than 3 or lower than 0.3, respectively, and a 95% confidence interval below 10% (criteria as [30]). In the present study the expression of *CXCR5 *and *CXCR4 *was 22.6 ± 0.7-fold higher and 3.5 ± 0.1-fold higher in RA tissue than in non-RA tissue, respectively. These results indicated that, of the chemokine receptors studied, *CXCR5 *was the most upregulated in RA (Table 3). The upregulation of *CXCR2 *could not be calculated for mathematical reasons because the signal intensity of *CXCR2 *in non-RA tissue after correction for the background was zero. *CXCR2 *was only visible on the non-RA autoradiogram upon prolonged exposure, at which point the housekeeping genes were saturated and were therefore unsuitable for quantification purposes.

Out of the 33 chemokines present on the arrays, 29 of these ligands were visible on the RA membranes and 21 on the non-RA membranes (Fig. 1 and Table 3). These included *CXCL13*, *CXCL12*, *CXCL8*, *CXCL1-3 *and *CXCL5*, which are ligands for the chemokine receptors CXCR5, CXCR4 and CXCR2. Several chemokines were visible on RA microarrays but not on non-RA microarrays (namely *CXCL13*, *CCL21 *and *CCL24*), suggesting that these genes might be induced in the inflamed synovium. In contrast, there were no chemokine signals that were present on non-RA membranes and were absent on RA membranes. Where chemokine signals were detectable on RA and non-RA microarrays, it was possible to quantify the degree of upregulation or downregulation using the criteria described earlier for chemokine receptors. Of these chemokines, the following showed upregulation: *CCL18 *(4.5 ± 0.4-fold increase), *CXCL9 *(3.6 ± 0.1-fold increase), *CXCL5 *(3.5 ± 0.3-fold increase), and *CXCL8 *(3.3 ± 0.2-fold increase). No chemokines displayed a downregulation with a RA/non-RA ratio less than 0.3. The upregulation of *CXCL9 *in RA synovia is in agreement with the only microarray study of RA synovia, in which this chemokine was also shown to be increased [31]. In our study only five chemokines (*CXCL7*, *CCL13*, *CCL20*, *CCL17 *and *CCL25*) could not be detected at all, whether in RA or non-RA samples.

The rapid screening of several genes at once made array technology a very attractive method. Its use has revealed disadvantages, however, including the requirement for large amounts of RNA (which are not always available from human tissue biopsies), a susceptibility to experimental variability and a lack of standard optimum methods for statistical analysis [32]. Arrays also present the risk of cross-hybridization leading to false positive or negative results [31]. However, the array approach remains a valuable tool if the samples can be pooled and if it is used in conjunction with alternative methods such as RT-PCR.

### RT-PCR analysis of *CXCR5*

**Figure 2:**
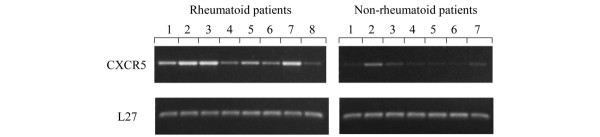
RT-PCR on rheumatoid arthritis and non-rheumatoid arthritis synovial tissue. CXCR5 RT-PCR products were separated on 0.8% agarose gels and stained with ethidium bromide. The reactions were performed on the individual RNA samples that were applied to the microarray membranes. The ribosomal RNA L27 was employed to normalize the amount of RNA used in each reaction.

To confirm the array results and to examine individual patients, RT-PCR was performed on the total RNA from each patient sample (Fig. 2). PCR primers were run through the BLAST program (available through the UK MRC HGMP-RC website: ) to ensure the gene specificity of the RT-PCR results and to exclude the possibility of cross-hybridization with other genes. Overall, CXCR5 RNA was more abundant in RA patients than in non-RA patients, confirming the microarray data. *CXCR5 *expression was detected in the synovia of all eight RA patients and showed some degree of patient-to-patient variation. The difference in *CXCR5 *expression between RA patients and non-RA patients was unlikely to be due to differences in the relative amount of cDNA produced by different RT reactions since the PCR reactions were normalized using the ribosomal gene *L27*. RT-PCR showed that the difference between RA patients and non-RA patients was less marked for *CXCR2 *and *CXCR4 *than for *CXCR5 *(data not shown).

### Immunohistochemistry

To identify the cell types expressing *CXCR5*, and since RNA expression and protein expression do not always correlate, the protein expression of this receptor and three specific cell markers (CD20, CD3 and CD68) was investigated by immunohistochemistry of paraffin-embedded sections.

**Figure 3:**
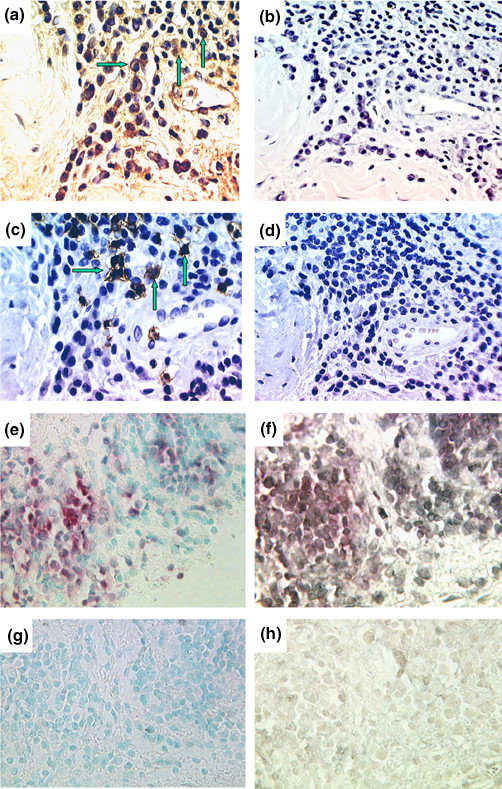
Immunohistochemistry of CXCR5 in lymphoid cell aggregates of rheumatoid arthritis synovia. Sections of rheumatoid synovium were treated with **(a) **CXCR5 antibody or **(b) **isotype control. Serial sections were treated with **(c) **anti-CD20 as a marker of B lymphocytes or **(d) **isotype control. Arrows indicate B lymphocytes expressing CXCR5. **(e) **Rheumatoid synovium treated with the T-cell marker anti-CD3 followed by alkaline phosphatase and Vector red substrate (methyl green counterstain). T cells stain a light red colour. **(f) **Serial section from the same synovial sample as (e) treated with anti-CD3, alkaline phosphatase and Vector red followed by anti-CXCR5, peroxidase and 3,3'-diaminobenzidine (DAB)-Nickel substrate (no counterstain used). T cells that express CXCR5 are stained dark red whereas cells expressing CXCR5 alone are grey–black in colour. **(g) **Control for (e), in which isotype-matched rabbit immunoglobulin (Ig) was used instead of anti-CD3. **(h) **Control for (f), in which isotype-matched rabbit and mouse Ig were applied instead of CD3 and CXCR5 antibodies (no counterstain used). Unless stated otherwise, DAB substrate was used. (a), (c) and (e)–(h) Original magnification, 420 ×; isotype controls (b) and (d) original magnification, 280 ×.

Seven out of eight RA patients presented substantial lymphoid follicles in their synovia. The specific cell markers CD20 and CD3 confirmed the presence of B cells and T cells, respectively, in these infiltrates. In every RA patient where lymphoid follicles occurred, CXCR5^+ ^cells were always present in these structures; this indicates a correlation between the expression of CXCR5 and the occurrence of lymphoid follicles. Serial sections indicated that CXCR5 was expressed by CD20^+ ^B cells (Fig. 3a,3c).

**Figure 4:**
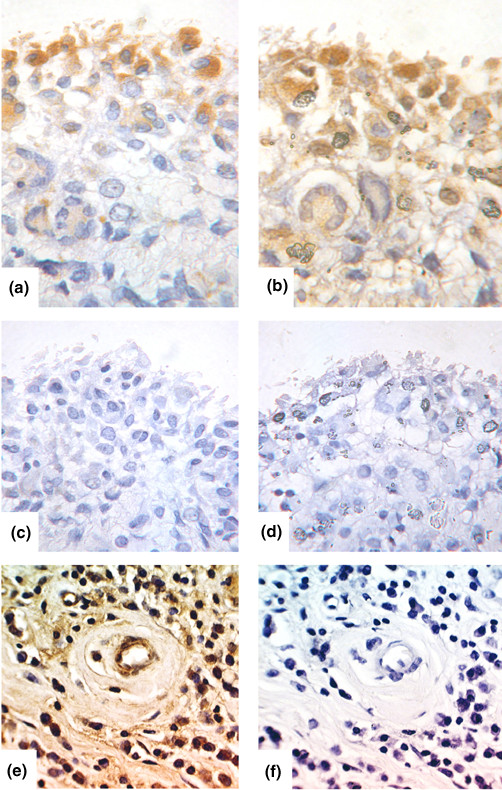
Immunohistochemistry of CXCR5 in the intima and postcapillary venules in rheumatoid arthritis synovia. **(a) **CD68^+ ^cells in the intima. **(b) **Serial section to (a) stained for CXCR5. Note the colocalization of CXCR5 and CD68 to the same group of cells. **(c) **and **(d) **Sections from the same region as (a) and (b), treated with isotype-matched control immunoglobulin instead of CD68 and CXCR5 antibodies, respectively. **(e) **Postcapillary venule positive for CXCR5 within a lymphoid aggregate. Labelling was revealed using 3,3'-diaminobenzidine substrate. **(f) **Isotype control for (e). (a), (b), (e) and (f) Original magnification, 420 ×; (c) and (d) original magnification, 350 ×.

It was not possible to colocalize CXCR5 and CD3 in serial sections, so a double-label immunohistochemistry technique was developed. Sections were treated with anti-CD3 followed by alkaline phosphatase and Vector red substrate. Anti-CXCR5 was added to the same sections, and the colour developed using peroxidase and DAB-Nickel. CD3 expression alone gave a light red colour (Fig. 3e) and CXCR5 expression alone produced a grey–black colour (Fig. 3f). Where these two proteins colocalized a dark red colour was obtained (Fig. 3f). Using this technique it was evident that in the RA synovium there was a population of CD3^+ ^T cells that expressed CXCR5 (Fig. 3f). These were localized exclusively in lymphoid follicles in the synovia of five out of the eight RA patients. The patient with diffuse infiltration was negative for CXCR5^+^/CD3^+ ^cells. Serial sections treated with anti-CXCR5 and the macrophage marker anti-CD68 suggested that CXCR5^+ ^cells in the intima included macrophages (Fig. 4a,4b). The endothelial cells of synovial postcapillary venules were positive for CXCR5 in the RA synovium (Fig. 4e).

**Figure 5:**
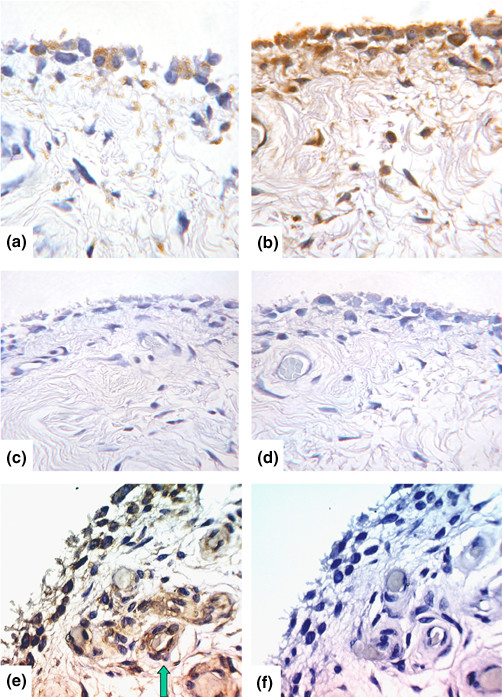
Immunohistochemistry of CXCR5 in non-rheumatoid arthritis synovia. **(a) **CD68 staining in the intimal layer. **(b) **Serial section to (a) treated with anti-CXCR5, showing that CXCR5^+ ^cells in the intimal layer included those also positive for CD68. **(c) **and **(d) **Sections from the same region as (a) and (b), treated with isotype-matched control immunoglobulin instead of CD68 and CXCR5 antibodies, respectively. **(e) **Subintimal postcapillary venule stains for CXCR5 expression (arrow). **(f) **Isotype-matched control for (e). Labelling was revealed using 3,3'-diaminobenzidine substrate. (a), (b), (e) and (f) Original magnification, 420 ×; (c) and (d) original magnification, 350 ×.

In non-RA tissue, CXCR5 was localized in the intima and endothelial cells (Fig. 5). Intimal cells were widely positive for CXCR5 and serial sections indicated that these included CD68^+ ^macrophage-like cells (Fig. 5a,5b). No lymphocytic infiltrates were present in these synovial samples due to their non-inflamed nature. Sections treated with CD20 and CD3 antibodies were negative, showing that no B cells or T cells could be detected. In non-RA tissue and RA tissue, fibroblasts were negative for CXCR5, as were neutrophils in RA synovia, indicating selectivity in the cell types expressing this receptor.

For all immunohistochemistry experiments in this study, the use of isotype-matched immunoglobulin controls or sera instead of primary antibodies resulted in negative staining of RA sections and non-RA sections (Figs 3b,3d,3g,3h, 4c,4d,4f and 5c,5d,5f).

### RT-PCR on isolated RA monocytes/macrophages

**Figure 6:**
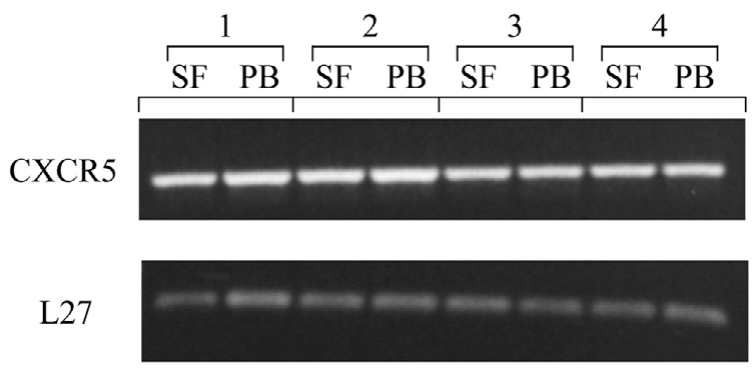
RT-PCR on monocytes/macrophages from peripheral blood (PB) and synovial fluid (SF). CXCR5 RT-PCR products were separated on 0.8% agarose gels and stained with ethidium bromide. The reactions were performed on four additional rheumatoid arthritis patients. The ribosomal RNA L27 was employed to normalize the amount of RNA used in each reaction.

To further investigate whether macrophages themselves are producing CXCR5 and to confirm the results of immunohistochemistry, we performed RT-PCRs on monocytes/macrophages isolated from the PB and SF of four additional RA patients (Fig. 6). *CXCR5 *was strongly expressed in all four samples and there was little difference between PB and SF.

## Discussion

The major finding of the present study is that CXCR5 is upregulated in the RA synovium. The cells expressing this chemokine receptor are B lymphocytes, T lymphocytes, macrophages and endothelial cells. The increased numbers of B lymphocytes, T lymphocytes and macrophages producing CXCR5 in the RA synovium are probably responsible for the increased expression of the receptor in this chronically inflamed tissue. The majority (seven out of eight) of the RA synovia included in this study contained substantial lymphoid aggregates but only one out of nine non-RA patients presented a very small infiltrate. These cell aggregates contained CD20^+ ^B cells that expressed CXCR5. The expression of CXCR5 has been reported in mature B cells and secondary lymphoid organs but as far as the authors are aware this is the first report of a chemokine receptor expressed by B cells in the RA synovium and its ectopic lymphoid structures.

Our findings are particularly interesting in view of the functional role of B cells in RA. This includes autoantibody production, antigen presentation, a role in lymphoid follicle and germinal centre formation, and the promising results of the anti-CD20 treatment in RA [33,34]. The microarrays showed that the mRNA for CXCL13, the only known ligand of CXCR5, was present in the RA synovium and not in the non-RA synovium. Furthermore, other reports have shown a CXCL13 message in RA synovia, together with its protein that localizes to follicular dendritic cells, endothelial cells and synovial fibroblasts, suggesting that these cells produce the chemokine [1,25]. Taken together with our data, this indicates that CXCR5 on B cells may be important in the recruitment of these cells into the RA synovium, in addition to their positioning and retention within the synovial infiltrates. In this regard, the role of CXCR5 on B cells in secondary lymphoid organs has been well documented [35,36]. CXCR5 guides B cells into the B-cell follicles and also directly promotes the recruitment of these cells into Peyer's patches via high endothelial venules [27,28,37,38]. In addition CXCR5-deficient mice exhibit impaired development of lymph nodes and Peyer's patches, and the tissue architecture of these organs is severely disturbed showing a lack of B-cell follicles [27,28].

Our double immunohistochemistry data indicate that there is a population of CXCR5^+^CD3^+ ^T cells present in the RA synovium. CXCR5^+ ^T cells have been shown in secondary lymphoid tissue where some of these cells localize within germinal centres [20,39], and it is proposed that CXCR5 enables them to enter B-cell follicles guided by CXCL13 [36]. Within these follicles they may provide B-cell help and have therefore been named follicular B helper T cells, since purified human tonsillar CD4^+^CXCR5^+ ^T cells efficiently stimulate the production of immunoglobulins by B cells [39,40]. These follicular B helper T cells are CD57^+^, whereas the majority of the CXCR5^+ ^T cells that are present in interfollicular and T-cell areas of the lymphoid tissue are CD57^- ^and are poor B-cell helpers [41]. Since lymphoid neogenesis occurs in the RA synovium it is possible that the CXCR5 expression on T cells as shown in the present study is involved in the positioning of these cells within the synovium and in providing B-cell help, although further studies are required to characterize this synovial T-cell population. Whether the two populations of CXCR5^+^CD57^+ ^and CXCR5^+^CD57^- ^T cells are present in the RA synovium and what their role could be is still unknown. However, CD57^+ ^T cells are reported to be present in the RA synovium and SF, where levels of this marker are elevated compared with controls [42,43]. Furthermore, an involvement of CD57^+ ^T cells has been shown in disease activity of RA [44].

Immunohistochemical experiments indicated that CD68^+ ^cells in the synovial intima express CXCR5. Intimal cells comprise two cell types: macrophage-like cells and fibroblast-like cells. In RA the former macrophage-like cells are numerous, comprising up to 80% of this cell layer [45]. It has been reported that in the RA synovium anti-CD68 reacts strongly with intimal macrophages, but fibroblasts also show some reactivity with this antibody [45]. Therefore, since macrophages are abundant in the RA intima and because of their strong reactivity with anti-CD68, it is likely that intimal macrophages are positive for CXCR5. In the normal non-RA intima, macrophages are positive for CD68 and fibroblasts are negative, making it more certain that macrophages express CXCR5 in this cell layer [45]. Consequently, RT-PCR was performed to verify that RA macrophages/monocytes can express *CXCR5*. The RT-PCR did indeed demonstrate CXCR5 mRNA in macrophages from RA SF, as well as PB monocytes from the same RA patients. Since the CXCR5 mRNA is expressed at similar levels in RA PB and RA SF it is suggested that the contribution of monocytes/macrophages to the upregulation of CXCR5 in the RA synovium is due to their increased number, rather than due to an increased abundance of CXCR5 transcripts per cell. CXCR5 mRNA has also been found in normal human PB monocytes by RT-PCR [21]. Studies by ourselves and other workers have shown that monocytes/macrophages express several other CXC chemokine receptors in RA, including CXCR1, CXCR2 and CXCR4 [15,16,46]. Furthermore, RA monocytes/macrophages express CC chemokine receptors such as CCR1, CCR2, CCR3 and CCR5 [14], illustrating their broad profile of chemokine receptor expression.

Endothelial cells are another cell type expressing CXCR5 in the synovium. There have been several reports of endothelial cells in the RA synovium expressing chemokine receptors, including CXCR3 and CXCR4, in addition to the Duffy antigen that is a non-signalling chemokine receptor [18,47-49]. In the RA synovium there is increased turnover of blood vessels with enhanced formation of new blood vessels together with enhanced vascular regression [50,51]. These mechanisms are regulated by the balance of angiogenic and angiostatic factors, and these factors include chemokines. Some chemokines are angiogenic (e.g. CXCL8, CXCL12, CCL1 and CCL2) and other chemokines are angiostatic (e.g. CXCL9 and CXCL10), and activation of their respective chemokine receptors results in the stimulation of or inhibition of endothelial cell proliferation [47,52-57]. CXCL13 has been shown to have an angiostatic function, inhibiting the angiogenic effects of FGF-2 on human umbilical vein endothelial cells [58]. In addition, the presence of CXCR5 in a variety of cultured human endothelial cells – from umbilical and saphenous veins, for example – may mediate the angiostatic effects of CXCL13 [58,59]. Our data showing the presence of CXCR5 on endothelial cells in the synovium and the presence of its ligand in this tissue [1,25]suggest that CXCR5 may play an angiostatic role in RA pathophysiology, although the angiostatic effects of CXCL13 could potentially be acting through CXCR3, which is also expressed by the RA synovial endothelium [48,60].

In the present study mRNA for other chemokine receptors were detected in the RA synovium, such as CXCR1, CXCR2, CXCR4, Bob, CCR1, CCR2a, CCR7, CCR9 and CX3CR1 (CXCR3 was not on the microarray). All of these showed variable degrees of increased mRNA expression in RA, although the upregulation was less compared with that of CXCR5. Several previous reports have shown the expression of chemokine receptors by leukocytes from RA joints. These have included CCR4–CCR6, CXCR3, CXCR4 and CX3CR1 by T lymphocytes [9,12,13,19] and CCR1–CCR5 and CXCR1–CXCR4 by monocytes/macrophages [14-16,18]. Such reports mainly focused on selected cell types and certain chemokine receptors. The present study took a different approach. Ours was primarily a whole-tissue study examining the mRNA expression of a wide range of chemokine receptors in RA and control synovia. While our study is in general accord with previous reports, differences may in part be due to the RA ST used. This tissue was highly infiltrated and, in all but one sample, had extensive lymphoid follicles bearing resemblance to those of secondary lymphoid organs. This feature may be responsible for the particular upregulation of the constitutive chemokine receptor CXCR5. In addition, our RA patients had long-standing disease (Table 1) and the patient sample may also have influenced the types of chemokine receptors expressed.

## Conclusion

Our study demonstrates the expression of CXCR5 on B cells, on T cells, on monocytes/macrophages and on endothelial cells in the RA synovium. The expression of a marker shared by cells that are known to play a central role in the process of chronic inflammation is of particular interest and suggests that targeting CXCR5 could provide a powerful new treatment for RA.

## Abbreviations

DAB = 3,3'-diaminobenzidine; H&E = haematoxylin and eosin; PB = peripheral blood; PBS = phosphate-buffered saline; PCR = polymerase chain reaction; RA = rheumatoid arthritis; RT = reverse transcription; SF = synovial fluid; ST = synovial tissue.

## Competing interests

The author(s) declare that they have no competing interests.

## Authors' contributions

CS carried out the microarray work, the RT-PCR and the double immunohistochemisty, and drafted the manuscript. AH carried out the single colour immunohistochemistry. AB isolated the peripheral blood and synovial fluid monocytes, and isolated the RNA after adhesion. BA participated in the design of the study. CB and MS collaborated on the study or coordinated the collection of samples in Birmingham, and contributed to the writing of the manuscript. JM conceived the study, and participated in its design and in the writing the manuscript. All authors read and approved the final manuscript.

## Figures and Tables

**Table 1:** Details of rheumatoid arthritis (RA) and non-RA patients

Patient (sex, age [years])	Diagnosis/pathology	Disease duration (years)	Medication
1 (male, 69)	RA	37	Auranofin, NSAID
2 (female, 41)	RA	23	NSAID
3 (female, 51)	RA	10	NSAID, analgesic
4 (female, 79)	RA	4	Methotrexate, NSAID, steroid
5 (female, 70)	RA	8	Penicillamine, steroid, NSAID
6 (female, 63)	RA + secondary osteoarthritis	39	Methotrexate, steroid, analgesic
7 (female, 33)	juvenile chronic arthritis	20	NSAID
8 (female, 66)	RA	38	NSAID, analgesic
1 (female, 50)	Articular cartilage damage	<1	NSAID, analgesic
2 (male, 44)	Meniscal tear	<1	-
3 (male, 42)	Meniscal tear and articular cartilage damage	4	-
4 (male, 60)	Meniscal degeneration	<1	-
5 (male, 52)	Articular cartilage degeneration	1	-
6 (male, 46)	Meniscal tear	<1	-
7 (male, 53)	Meniscal degeneration	<1	-
8 (male, 38)	Meniscal tear	1	Steroid
9 (male, 43)	Articular cartilage degeneration	<1	-
1 (female, 91)	RA	<1	Analgesic
2 (female, 56)	RA	<1	NSAID, steroid, methotrexate
3 (male, 67)	RA		-
4 (male, 67)	RA	4	Analgesic, NSAID, methotrexate

Synovia were obtained from eight RA patients and nine non-RA patients.

Monocytes/macrophages from peripheral blood/synovial fluid were obtained from the last four patients. NSAID, non-steroidal anti-inflammatory drug.

**Table 2:** Sequences of the primers used for RT-PCR

mRNA	Product	Sequence	Size (base pairs)	Accession number
L27	Forward	5'-GACGCAAAGCTGTCATCGTG-3'	344	BC007273
	Reverse	5'-GCAGTTTCTGGAAGAACCAC-3'		
CXCR5	Forward	5'-TGA CCT GAG GAA GCG TGA AG-3'	639	NM001716
	Reverse	5'-CGT GAA GAC ACT CTC ACG TG-3'		

**Table 3:** Chemokine and chemokine receptor expression data analysis

Gene	RA	Non-RA	Regulation (ratio RA/non-RA)
**Receptors**			
Array column 3 (Fig. 1, C3)			
CCR1	0.050	0.023	Up (2.2 ± 0.2)
CCR2a	0.031	0.012 ○●	Up (2.7 ± 0.2)
CCR2b	0.000 ○	0.000 ○	Not visible (NA)
CCR3	0.000 ○	0.000 ○	Not visible (NA)
CCR4	0.003 ○	0.000 ○	Not visible (NA)
CCR5	0.042	0.000 ○●	Up (NA)
CCR6	0.001 ○	0.000 ○	Not visible (NA)
CCR7	0.022 ○●	0.011 ○	Not visible (2.5 ± 0.9)
CCR9	0.001 ○●	0.000 ○	Not visible (NA)
CX3CR1	0.028 ○●	0.019 ○●	Not visible (1.5 ± 0.1)
CXCR1	0.036 ○●	0.008 ○●	Not visible (9.6 ± 5.6)
CXCR2	0.651	0.000 ○●	Up (NA)
CXCR4	0.190	0.055	Up (3.5 ± 0.1)
CXCR5	1.328	0.059	Up (22.6 ± 0.7)
CXCR6 (STRL33)	0.016	0.025	Not visible (0.7 ± 0.0)
Bob	0.034	0.000 ○	Up (NA)
**Chemokines**			
Array column 1 (Fig. 1, C1)			
CCL21 (6Ckine)	0.030	0.019 ○	Up (1.6 ± 0.1)
CXCL13 (BLC/BCA-1)	0.052	0.020 ○	Up (2.8 ± 0.4)
CXCL10 (IP-10)	0.031	0.019 ○●	Up (1.6 ± 0.1)
CXCL5 (ENA-78)	0.050	0.015 ○●	Up (3.5 ± 0.3)
CCL11 (eotaxin)	0.050	0.052	Not visible (1.0 ± 0.0)
CCL24 (eotaxin-2)	0.032	0.013 ○	Up (2.5 ± 0.3)
CX3CL1 (fractalkine)	0.030 ○●	0.012 ○	Not visible (2.5 ± 0.2)
CXCL1 (GRO-α)	0.090	0.057	Up (1.6 ± 0.0)
CXCL2 (GRO-β)	0.151	0.101	Up (1.5 ± 0.0)
CXCL3 (GRO-γ)	0.101	0.055	Up (1.8 ± 0.0)
CCL14 (HCC-1)	0.202	0.296	Down (0.7 ± 0.0)
CCL16 (HCC-4)	0.006 ○●	0.000 ○	Not visible (NA)
CCL1 (I-309)	0.007 ○●	0.000 ○	Not visible (NA)
CXCL8 (IL-8)	0.086	0.026	Up (3.3 ± 0.2)
CXCL7 (LDGF)	0.005 ○	0.000 ○	Not visible (NA)
CCL15 (MIP-1δ)	0.009 ○●	0.008 ○	Not visible (11 ± 13)
XCL1 (lymphotactin)	0.048	0.031	Up (1.6 ± 0.0)
CCL2 (MCP-1)	0.482	0.545	Not visible (0.9 ± 0.0)
CCL8 (MCP-2)	0.053	0.062	Not visible(0.9 ± 0.0)
CCL7 (MCP-3)	0.066	0.047	Up (1.4 ± 0.1)
CCL13 (MCP-4)	0.000 ○	0.002 ○	Not visible (0.0)
CCL22 (MDC)	0.000 ○●	0.008 ○	Not visible (0.0)
Array column 2 (Fig. 1, C2)			
Midkine	0.299	0.155	Up (1.9 ± 0.0)
CXCL9 (MIG)	0.166	0.047	Up (3.6 ± 0.1)
CCL3 (MIP-1α)	0.040	0.042	Not visible (1.0 ± 0.0)
CCL4 (MIP-1β)	0.023	0.018 ○●	Up (1.3 ± 0.2)
CCL20 (MIP-3α)	0.000 ○	0.013 ○	Not visible(0.0)
CCL19 (MIP-3β)	0.023	0.042	Not visible (0.6 ± 0.0)
CCL23 (MPIF-1)	0.039	0.042	Up (0.9 ± 0.0)
CCL18 (PARC)	0.146	0.033	Up (4.5 ± 0.4)
CCL5 (RANTES)	0.037	0.031	Up (1.2 ± 0.1)
CXCL12 (SDF-1)	0.409	0.573	Down (0.7 ± 0.0)
CCL17 (TARC)	0.000 ○	0.000 ○	Not visible (NA)
CCL25 (TECK)	0.000 ○	0.000 ○	Not visible (NA)

Following hybridization to labelled mRNA extracted from rheumatoid arthritis (RA) and non-RA synovia, a pair of array membranes was autoradiographed for varying lengths of time. The autoradiograms were scanned and analysed with the ArrayVision software (version 6.0; Imaging Research Inc., Haverhill, UK). For each RA/non-RA pair the housekeeping genes on the membranes showed very similar intensities, were not saturated and were used to normalize the data. The analysis measured the 'volume' of each spot (i.e. the density value of each spot multiplied by its area). The background was measured using the 'corners between spots' protocol of the software and was deducted from the 'volumes'. The ratio of RA synovia versus non-RA synovia was also calculated for each spot. The analysis was repeated eight times for each pair of autoradiograms, providing 16 values for each gene (each gene is spotted in duplicate) on each pair. Figures in the columns RA, non-RA and ratio RA/non-RA represent the average of 16 values. For each average ratio the 95% confidence level was calculated, and the results presented are those from the autoradiogram pair giving the smallest variation. ○, spot was not visible by eye on the corresponding autoradiogram; ●, spot was visible after prolonged exposure. The mRNA regulation of RA versus non-RA as observed by eye at the time point used for quantification is indicated by not visible, up or down. NA, ratio could not be calculated due to the presence of zero values. The recent systematic nomenclature of chemokines is used, with the former names in parentheses. The order of the genes presented is the same as that appearing on the microarray in Fig. 1.
